# Supplementary material for: Should you become a leader in online collaborative learning? Impact of assigned leadership on learning behaviors, outcomes, and perceptions
Source: PLoS One. 2022 Apr 7;17(4):e0266653. doi: 10.1371/journal.pone.0266653 (PMC8989189; doi:10.1371/journal.pone.0266653)
Supplement: S1 Appendix — (DOCX) [file pone.0266653.s001.docx]

**S1 Appendix. Learning experience questionnaire items.**

**Part 1: Please fill out the following information**

1. Your name is
2. Your birth sex is: ○ Man ○ Woman
3. Your role in the group is: ○ Leader ○ Member

**Part 2: Please evaluate your learning experience in the blended course (1: strongly disagree, 2: disagree, 3: neutral, 4: agree, 5: strongly agree)**

1. I had a great learning experience in this unit/semester.
2. I took an active participation in group discussion.
3. I have made my due contribution to my group in this unit/semester.
4. I have promoted collaborative learning in my group.
5. I think our group discussion had sufficient depth.
6. I have benefited from discussing with peers in my group.
7. The collaboration in my group was pleasant in this unit/semester.
8. I think the task assignment in this unit/semester was fair for everyone in the group.
9. I look forward to learning the next unit/I will recommend this course to other students.
