# Supplementary material for: Should you become a leader in online collaborative learning? Impact of assigned leadership on learning behaviors, outcomes, and perceptions
Source: PLoS One. 2022 Apr 7;17(4):e0266653. doi: 10.1371/journal.pone.0266653 (PMC8989189; doi:10.1371/journal.pone.0266653)
Supplement: Attachment — Submitted filename: response to reviewer.docx [file pone.0266653.s003.docx]

Response to Reviewers

We greatly appreciate this opportunity to revise our manuscript and address the potential issues identified by the editor and the reviewer. We are very grateful for their careful review and insightful feedback. Our point-by-point responses to the individual comments made by each reviewer are listed in below. Please also refer to the tracked changes for detailed revisions.

## Editor comments

When submitting your revision, we need you to address these additional requirements.

**Comment E1**: Please ensure that your manuscript meets PLOS ONE's style requirements, including those for file naming.

**Response E1:** To ensure that our manuscript format and style meet the journal requirement, we have sent our manuscript to a professional editing service for format check and edits. Please see the certificate in below:

**Comment E2:** Please change "female” or "male" to "woman” or "man" as appropriate, when used as a noun

**Response E2:** Change accordingly.

**Comment E3:** *In order to improve reporting, in your methods section, please provide additional information about the demographic details of your participants.

**Response E2:** We have added demographic details of the participants in the methods section, including gender, age, academic program. See below for the revision:

Line 151-155: A total of 94 graduate students from a research university in central China participated in this quasi-experimental study during the 2019 fall semester. The participant group was predominantly female with 86 female students and 8 male students. They were all first-year students admitted to a graduate program of educational technology, with an average age of 23.6 (ranging from 21 to 25)…

**Comment E4:** Please include your full ethics statement in the ‘Methods’ section of your manuscript file. In your statement, please include the full name of the IRB or ethics committee who approved or waived your study, as well as whether or not you obtained informed written or verbal consent. If consent was waived for your study, please include this information in your statement as well.

**Response E4:** Ethics Statement was added to the Methods section. See below:

Line 142-149: The research study was conducted in accordance with the ethical standards of the Helsinki Declaration. The research procedures and instruments were reviewed and approved by the Institutional Review Board of Central China Normal University (CCNU-IRB-201909021, approved on 2019/09/16). Written informed consent forms were obtained from all participants before the study. All participants were made aware that their participation in the study was voluntary, and their personal identifiable information would be kept anonymous at all publications and presentations. Participants can withdraw from the research study anytime, without penalty.

## Reviewer Comments

**Comment R1:** This study tests the effect of assigned leadership on online behaviors, outcomes, and perceptions, using data of 94 student-course observations from a China's university. The author needs to explain why the course is suitable for investigating this topic.

**Response R1:** We added the rationale for our course selection. See below:

Line 155-160: The study was implemented in a blended course called instructional design and case analysis, which lasted for 12 academic weeks. Students were required to apply teaching and learning theories to solve authentic instructional problems through group activities such as case analysis, online discussion, and collective reflection. This course was selected for investigating the research topic due to its complexity and ill-structured nature, which are known to promote collaborative learning and group interaction [4,14].

**Comment R2:** The students who more skewed to the female could be one of the limitations. I suggest the author use more student-course observations to overcome gender bias.

**Response R2:** We agree that the participants were skewed to female and the results might be gender-bias as a result. We added such discussion in the section of Limitations and future research. However, we would argue that the predominantly female participant group might boost the internal validity of the research findings as the extraneous variable of gender is controlled somehow…We assume that gender distributions (e.g., all-male, all-female, and co-ed groups) might lead to different group dynamics, which can extraneously affect the effect of assigned leadership. This can be further investigated in the future. As for the student-course observation, most of the collaborative learning activities occurred in the online learning environment, making it impossible to “actually” see them in action…Rather, we analyzed the learning behaviors in terms of forum analytics and posted content. Please see our revisions in below:

Line 423-425: Second, the participants were predominantly female students from a single course, which undermines the generalizability of the study results to other CSCL contexts as the results might be gender-biased and driven by course-specific knowledge.

Line 429-431: Consequently, we recommend future research to investigate the impact of assigned leadership in varied CSCL contexts (e.g., co-ed groups from various disciplinary domains) with more rigorous design and diversified empirical data.

**Comment R3:** The results may be driven by the course-specific knowledge. I suggest the author should employ more comprehensive data including all courses at the university for each student.

**Response R3:** Thank you for this suggestion. We agree that the research findings from one specific course might suffer from insufficient generalizability, and thus replicate studies in other disciplinary courses are needed in the future. However, collecting and analyzing all-course learning analytics for each student is not feasible in the present study, for three reasons.

First, the status of assigned leader role is the dependent variable investigated in this quasi-experiment. While random assignment of leader role is feasible in this particular course taught by the first author, it might not be possible in other courses since not all courses utilized the pedagogy of collaborative learning and the grouping strategies.

Second, students might assume different roles (e.g., leader, member, etc.) in different courses, making it impossible to attribute the comprehensive all-course learning analytics to a specific instructional role.

Third, for any experimental research study, it is essential to control for the extraneous variables so that the causal relationship regarding the experiment condition can be established. If we include data from multiple courses, it will be extremely difficult to control for the extraneous variables. For example, factors such as instructor, learning tasks, peers, and learning motivation might be drastically different across different courses, posing internal validity threats to the research conclusions.

We do, however, acknowledge the limitation of implementing the research study in a single course, and provided discussion in the section of Limitations and future research.

**Comment R4:** It would be useful to further investigate and discuss the relationship between assigned leaders, emerged leaders, and distributed leaders.

**Response R4:** We added a new paragraph in the section of Discussion and perspectives to discuss the relationship among the three leaderships. Please see below:

Line 387-397: Consistent with the existing literature [10,27,52], instances of emerged and distributed leadership were also reported in this study, and two types of relationship between assigned and non-assigned leadership roles were identified. The first type is substitution, where an assigned leader failed to fulfill leading responsibilities and was replaced by an emerged leader during the collaborative learning process. The phenomenon of substitution is expectable with random assignment of leader role, where unwilling or incapable students have equal chance to get elected as group leaders. The second type is distribution, where the emergence of on-assigned leadership was more contingent with the attributes of a particular group member (e.g., academic achievement, personality, etc.) rather than the involvement of the assigned leader. Consequently, distributed leadership should not be viewed as a social status, but rather a preferred relationship between assigned and emerged leadership.

**Comment R5:** I would also encourage you to engage a professional copyeditor to polish the writing of the paper.

**Response R5:** Thank you for the suggestion. We have sent our manuscript to a professional copyeditor for premium editing. We also carefully proofread the manuscript for another time during the revision. Hopefully, all language issues were fixed. Please see the editing certificate in below:
