# Supplementary material for: Should you become a leader in online collaborative learning? Impact of assigned leadership on learning behaviors, outcomes, and perceptions
Source: PLoS One. 2022 Apr 7;17(4):e0266653. doi: 10.1371/journal.pone.0266653 (PMC8989189; doi:10.1371/journal.pone.0266653)
Supplement: Attachment — Submitted filename: referee report PLOS ONE.docx [file pone.0266653.s002.docx]

Below are comments for the authors to consider.

Comments:

1. This study tests the effect of assigned leadership on online behaviors, outcomes, and perceptions, using data of 94 student-course observations from a China's university. The author needs to explain why the course is suitable for investigating this topic.

2. The students who more skewed to the female is could be one of the limitations. I suggest the author use more student-course observations to overcome gender bias.

3. The results may be driven by the course-specific knowledge. I suggest the author should employ more comprehensive data including all courses at the university for each student.

4. It wold be useful to further investigate and discuss the relationship between assigned leaders, emerged leaders, and distributed leaders.

5. I would also encourage you to engage a professional copyeditor to polish the writing of the paper.
